# Improving Mental Skills in Precision Sports by Using Neurofeedback Training: A Narrative Review

**DOI:** 10.3390/sports12030070

**Published:** 2024-02-29

**Authors:** Stefano Corrado, Beatrice Tosti, Stefania Mancone, Tommaso Di Libero, Angelo Rodio, Alexandro Andrade, Pierluigi Diotaiuti

**Affiliations:** 1 Department of Human Sciences, Society and Health, University of Cassino and Southern Lazio, 03043 Cassino, Italy; stefano.corrado@unicas.it (S.C.); beatrice.tosti@unicas.it (B.T.); s.mancone@unicas.it (S.M.); tommaso.dilibero@unicas.it (T.D.L.); a.rodio@unicas.it (A.R.); 2 Health and Sports Science Center, Department of Physical Education, CEFID, Santa Catarina State University, Florianópolis 88035-901, Brazil; alexandro.andrade@udesc.br

**Keywords:** sport shooting, archery, golf putting, neurofeedback, neuromodulation, brain wave patterns, anxiety reduction, concentration, cognitive control

## Abstract

Primary objectives of neurofeedback training (NFT) are to improve concentration, stress and anxiety management, and performance optimisation. This narrative review examines the role of NFT as a tool to improve mental and cognitive skills of target shooting and archery athletes. Current research discusses how neurofeedback training can act on brain waves by influencing specific EEG frequency bands in order to improve cognitive flexibility. This contribution reports studies that have applied neurofeedback protocols in precision disciplines such as archery and shooting. The results of the studies considered showed that neurofeedback can lead to faster reaction times, more sustained attention, and better emotion management, contributing significantly to athletes’ performance. Furthermore, it is emphasised that neurofeedback could be combined with other techniques such as motor imagination to maximise effectiveness in precision sports training. This review emphasises the importance of future studies that focus on the integration of neurofeedback with biofeedback in neuromodulation protocols. Current perspectives and limitations of research in this area are also indicated. Neuromodulation by means of neurofeedback represents a promising strategy to improve the overall mental and cognitive abilities of target shooting and archery athletes with an interesting potential for high-level performance. Future research should focus on integrated approaches and customised protocols to optimise the use of neurofeedback in a precision sports context.

## 1. Introduction

The psychological and cognitive components of athletic performance have garnered significant interest within the realms of sports science and psychology. Precision sports, such as archery and shooting, demand not only physical skill but also high levels of mental concentration, emotional control, and cognitive flexibility. These mental attributes are critical, as they directly influence an athlete’s ability to perform under pressure, maintain focus over prolonged periods, and manage the stress and anxiety that competitive environments engender. Traditional training methodologies in these sports have primarily focused on the physical aspects of performance enhancement, often overlooking the substantial impact of psychological and cognitive training.

In shooting sports, mental qualities that affect attentional control and emotional states are constantly required, especially in the phases leading up to shooting at a target [1]. It has been shown that adequate emotional control conducted by the athlete together with high mental efficiency can improve performance in target shooting [2]. Archery as well as shooting are sports where the psychological aspects have such a weighted effect on performance that they are referred to as mental sports. For this reason, they require constant and adequate psychophysiological training [3]. Taha and collaborators conducted a study in which they sought to define the mental abilities of archers, establishing that a good archer must exhibit adequate mental concentration, optimally manage stress and anxiety, and be able to control internal emotional states [4]. A lack of full emotional and mental control can lead to states of mental blockage, caused by actual panic attacks on the technical conduct of the shot [5,6].

Recent advancements in neuroscience and sports psychology have underscored the potential of neurofeedback training (NFT) as a tool for cognitive and psychological enhancement. NFT, a form of biofeedback that uses real-time displays of electroencephalography (EEG) to teach the self-regulation of brain functions, has emerged as a promising approach for improving the mental skills that are essential for high-level performance in precision sports [7]. Through the modulation of specific EEG frequency bands, NFT aims to enhance cognitive processes such as attentional focus, stress management, and emotional regulation—key components that can determine the fine line between success and failure in sports competitions [8,9]. In addressing the multifaceted nature of neurofeedback (NFT) within the realm of sports performance enhancement, it is imperative to acknowledge that NFT extends beyond the commonly discussed electroencephalogram (EEG) applications. A critical component of NFT, especially pertinent in precision sports such as shooting, encompasses heart rate variability (HRV) and respiratory training—key physiological aspects that significantly impact performance. Unlike EEG, which primarily focuses on brain wave training, HRV and respiratory training within the NFT framework aim to optimise athletes’ physiological control, enhancing their ability to maintain calmness and precision under pressure. Studies have demonstrated the efficacy of incorporating HRV and respiratory training in improving athletes’ performance, underscoring the importance of a comprehensive approach to neurofeedback that includes these physiological feedback mechanisms [10,11,12]. Thus, while EEG-based neurofeedback plays a significant role in cognitive enhancement, the broader spectrum of NFT, encompassing heart rate and breathing control, is indispensable in sports science, offering a holistic approach to athlete training and performance optimization.

EEG-based neurofeedback involves the placement of electrodes on the scalp, which serve to detect EEG signals from various cortical areas [13]. The subject, through operant conditioning, manipulates these signals by receiving instantaneous feedback. This feedback is provided in auditory, visual, or a combined audio-visual form, enabling the individual to influence and modify brain wave frequency bands in the targeted regions of interest [14].

**Figure 1:**
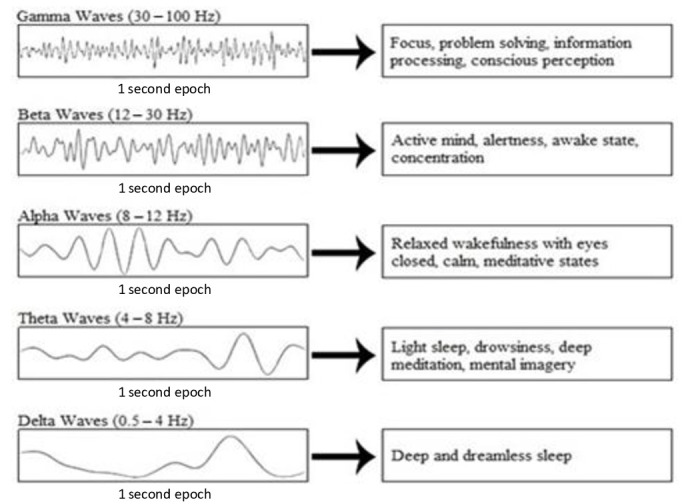
EEG oscillations and their corresponding cognitive processes.

EEG-based neurofeedback training operates through the modulation of EEG frequency bands, where delta (1–4 Hz) is recognised as the slowest wave associated with deep sleep, followed by theta (4–8 Hz), alpha (8–12 Hz), beta (12–30 Hz), and gamma (30 Hz and above) bands, each associated with progressively higher cognitive functions. In the context of enhancing cognitive flexibility and performance in precision sports, neurofeedback training specifically targets the optimization of these frequency bands. For instance, enhancing alpha and theta waves can improve relaxation and concentration, crucial for precision sports like archery and shooting, while the modulation of beta waves can enhance alertness and decision making [15]. Figure 1 provides a detailed visualization of EEG oscillations and their corresponding cognitive processes, offering insights into the specific brain wave patterns associated with different cognitive states and their implications for neurofeedback interventions in sports performance enhancement.

Therefore, cortical oscillations can be used to understand the involvement of the cerebral cortex during the performance of different tasks. Initially, the changes are short-lived, but gradually, they become more long-lasting, and it has been shown that, with continuous feedback, brain wave patterns can be retrained to improve flexibility and cognitive control [16].

Neurofeedback training extends beyond EEG, utilising fMRI (Functional Magnetic Resonance Imaging), fNIRS (functional near-infrared spectroscopy), and other methods to collect neural signals, offering insights into the brain’s functional dynamics. This comprehensive approach enables the targeted enhancement of cognitive flexibility and control—crucial for adapting strategies under varied conditions and orchestrating actions aligned with goals. In emphasising the role of neurofeedback training in sports, it is crucial to highlight its contribution to enhancing mental agility and focus. Such improvements are particularly vital for athletes in precision sports, such as shooting and archery, where NFT’s advanced brain training techniques play a key role in optimising performance.

Building on the foundational understanding of NFT’s application in sports, it is essential to delve into its scientific underpinnings and the specific cognitive functions it aims to enhance in athletes participating in precision sports. The application of neurofeedback training in the realm of precision sports is grounded in its ability to enhance specific cognitive functions critical for peak performance. NFT operates by allowing athletes to gain real-time insights into their brain wave patterns, facilitating the development of strategies to regulate these patterns for improved focus, attention, and emotional regulation. Scientifically, NFT’s efficacy lies in its targeted approach to enhancing neuroplasticity—the brain’s ability to reorganise itself by forming new neural connections. This is particularly relevant in sports such as shooting and archery, where minute lapses in concentration can significantly impact accuracy and outcomes. In the context of precision sports, cognitive functions such as sustained attention, emotional regulation, and visuospatial processing are critical for optimal performance. NFT specifically targets these cognitive domains by training athletes to enhance their neural activity patterns that correlate with these functions.

Research has demonstrated that NFT can effectively improve attentional control, a cognitive function that enables athletes to maintain focus on their task while ignoring distractions. This is crucial in precision sports where sustained attention and mental steadiness are paramount [17]. Moreover, NFT has been shown to enhance emotional regulation, aiding athletes in managing anxiety and pressure, conditions often encountered in competitive sports environments [18]. These improvements in cognitive functions contribute directly to an athlete’s ability to perform consistently under stress, making NFT a valuable tool in the mental training regimen of precision sports athletes.

The scientific basis for NFT’s role in sports performance enhancement is further supported by studies utilising neuroimaging techniques such as fMRI and fNIRS, which provide empirical evidence of changes in brain activity patterns associated with successful NFT interventions. These changes often correlate with improvements in cognitive functions, underscoring NFT’s potential to influence brain areas responsible for attention, emotional regulation, and decision-making processes that are crucial in precision sports [19].

Incorporating NFT into the training programs of athletes in precision sports thus offers a science-backed approach to cultivating the mental skills necessary for excellence. By focusing on specific cognitive functions that underpin precision and accuracy, NFT equips athletes with the mental agility and focus required to excel in their respective disciplines.

Despite the growing interest and preliminary evidence supporting the effectiveness of NFT in sports, the literature remains fragmented with respect to its application and outcomes in precision sports. This narrative review seeks to consolidate the current state of knowledge regarding the use of NFT to enhance mental and cognitive skills in precision sports athletes. By doing so, it aims to provide a comprehensive overview of the methodologies, outcomes, and theoretical underpinnings of NFT in this context.

Specific objectives of this review:

1. To synthesise the existing research on the application of NFT in improving cognitive and mental skills in athletes, with a specific focus on precision sports such as archery and shooting;
2. To evaluate the effectiveness of NFT protocols in enhancing performance outcomes in precision sports by reviewing and analysing the outcomes of relevant studies;
3. To identify the theoretical mechanisms through which NFT may influence cognitive and psychological processes relevant to sports performance;
4. To discuss the current limitations and future directions of research in the application of NFT in sports, with an emphasis on precision disciplines.

The burgeoning interest in neurofeedback training within the realm of sports science is predicated on its potential to harness the brain’s plasticity, thereby enhancing cognitive and mental capabilities that are critical for athletic performance. This narrative review delineates three pivotal aspects of NFT application in sports: general performance enhancement; specific improvements in target-precision disciplines such as archery and shooting; and technological advancements for neuromodulation in sports. Therefore, three sections addressing these topics have been included in the text.

The inclusion of a Section 2 is grounded in the foundational premise that optimal athletic performance transcends physical capabilities, deeply entwined with cognitive and psychological resilience. Neurofeedback training offers a biofeedback mechanism that enables athletes to modulate their brain wave patterns consciously, facilitating improvements in focus, anxiety management, and stress response—qualities universally beneficial across all sports disciplines. By presenting this section, we aim to lay a comprehensive framework illustrating how NFT can be a game-changer in enhancing athletes’ overall performance, setting the stage for a deeper exploration into its role in precision sports.

The rationale for focusing on “Neurofeedback and Target-Precision Sports” emerges from the unique demands that these sports place on athletes, including heightened levels of concentration, fine motor control, and emotional regulation during critical moments of performance. Precision sports such as archery, shooting, and golf require a delicate balance between cognitive and physical skills to achieve accuracy and consistency. This segment is dedicated to unravelling the specificity of NFT applications in these disciplines, highlighting empirical evidence and theoretical underpinnings that suggest that neurofeedback can significantly impact athletes’ ability to maintain composure, focus, and precision under pressure. It underscores the potential of NFT to fine-tune the mental skills that dictate success in target-precision sports, offering insights into tailored training protocols that leverage neuroplasticity for sporting excellence.

The Section 5 encapsulates the intersection of cutting-edge technology with the domains of neuroscience and sports performance. It emphasises the exponential growth in technological innovations that have significantly enhanced the precision, customization, and accessibility of neuromodulation and biofeedback tools. These advancements enable the development of highly tailored training programs that cater to the unique physiological and psychological profiles of athletes, promoting optimal performance outcomes in precision sports. The democratization of these technologies has broadened their availability, allowing athletes at various levels to leverage sophisticated training aids that were once reserved for the elite. Integration with traditional training regimens offers a holistic approach to athlete development, addressing mental, emotional, and physical aspects of performance.

By setting a clear scientific background and delineating these objectives, this review aims to bridge the gap between NFT research and its practical applications in precision sports. It endeavours to provide athletes, coaches, and sports scientists with insights into the potential benefits of incorporating NFT into training regimens, thereby fostering a holistic approach to performance enhancement that encompasses both the physical and mental facets of athletic achievement.

## 2. Neurofeedback and Sport Performance

In recent years, the application of NFT in enhancing sport performance has garnered significant interest within the sports science community. Neurofeedback training, which involves the operant conditioning of brain waves, has been shown to offer substantial benefits across a range of sports, especially those requiring high precision and concentration such as archery, shooting, golf, and even team sports like soccer and basketball [20,21].

Prominent studies have investigated aspects involving concentration, intended as being able to recognise when one is off task, trying to group together any thoughts/feelings that avoid interrupting attention. Greater concentration produces faster reaction times. For this reason, Bandmeyer and Delorme, in 2020, used neurofeedback to increase theta power in the frontal area, with the aim of training meditation in attentional tasks and achieving fast reaction times [22].

In line with this research, Maszczyk and colleagues, in 2020, observed an improvement in reaction time in judo using an NFT protocol through which they were able to maintain an optimal balance between fast wave (beta) and slow wave (theta) activity, which was found to be associated with concentration [23]. Harvey and colleagues introduced this technique as an integral part of a training programme for a Canadian national skating team heading to the Olympic Games, and the results showed an improvement in reaction time [24].

Another important psychological construct in sport is sustained attention, i.e., the ability to focus on an action for extended periods of time and associated with the top-down control of theta activity [25]. A specific NFT for this function shows positive correlations with the control of complex actions, such as putting in golf [25,26,27].

A further aspect associated with improved sports performance sees the involvement of neurofeedback in stress and anxiety management. Athletes and coaches claim that NFT can provide effective stress management, through self-awareness and self-regulation, resulting in improved performance [28].

Excessive arousal and state anxiety are further contributors to negative performance, which, in addition to generating noticeable incoordination caused by increased muscle tension, lead to more distracting effects and irrelevant thoughts. However, it has long been known that an alpha wave-targeted neurofeedback intervention improves not only the quality of one’s emotional state, but also aspects such as stress and anxiety and functions such as memory [29].

Alpha-band training in the right hemisphere of the sensory cortex can induce a state of relaxation. Domingos in 2021 noted that alpha training can improve heart rate variability in athletes who received NFT for four consecutive weeks [30].

In precision sports like archery and shooting, sporting performance is intricately tied to cognitive abilities, notably cognitive flexibility, alongside stress management and self-regulation. These sports demand the athlete’s capacity to adapt quickly to changing conditions and maintain concentration under pressure. Cognitive flexibility enables athletes to switch strategies effectively, while stress management and self-regulation help in maintaining focus and emotional balance.

To ensure the application of neurofeedback training (NFT) protocols based on the latest scientific evidence and international best practices, continuous training of NFT operators is crucial. The effectiveness of NFT is evident in various studies. For instance, research by Zoefel et al. (2011) demonstrated that NFT targeting specific EEG frequency bands, such as the upper alpha band, can enhance cognitive performance [31]. Similarly, Gong et al. (2021) emphasised the importance of verifying NFT’s training effects on behavioural outcomes and its correlation with learning, highlighting the need for evidence-based practices in NFT [32]. Su et al. (2021) discussed the importance of reducing training sessions and achieving effective advantages in NFT, emphasising the need for continuous improvement and adherence to the latest evidence-based approaches [33]. Moreover, the study by Schabus et al. (2017) highlighted the significance of double-blind placebo-controlled neurofeedback studies, emphasising the importance of rigorous scientific evidence in validating NFT interventions [34]. Additionally, Enriquez-Geppert et al. (2019) pointed out the need for current evidence and practice in NFT for ADHD, indicating the importance of aligning NFT protocols with the latest scientific understanding [35]. Renton et al. (2015) stressed the need for systematic reviews to understand the overall impact of NFT, highlighting the importance of evidence-based approaches in NFT interventions [36].

## 3. Methodology

Our selection of studies for this review was guided by their relevance to the central theme of neurofeedback in precision sports. The narrative approach was chosen due to the exploratory nature of this field, which encompasses diverse methodologies and findings, making a systematic review impractical at this stage. We recognise the potential for selection bias that is inherent in narrative reviews. To mitigate this, we have strived for a balanced representation of the literature, including studies with both positive and negative outcomes. This approach ensures a more comprehensive understanding of the effectiveness of neurofeedback in precision sports. In presenting our findings, we systematically analysed and summarised key outcomes from each study. This included a range of variables such as study design, participant demographics, neurofeedback protocols used, and performance outcomes. To ensure a comprehensive and unbiased overview, we conducted an extensive literature search focusing on neurofeedback training in precision sports. Our methodology included searching databases such as PubMed, Scopus, and the Web of Science using keywords like “neurofeedback”, “precision sports”, “target shooting”, and “archery”. We meticulously selected studies published between 1990 and 2023, applying inclusion criteria that focused on the application of neurofeedback in sports performance. Exclusion criteria were studies not directly related to neurofeedback or precision sports. In addition to delimiting the study to precision shooting disciplines, the search for articles considered the following subject areas overall: (1) neurofeedback training and cognitive performance (focusing on the impact of neurofeedback training on cognitive functions such as attention, concentration, and reaction time); (2) physiological aspects in sports performance (research on the physiological responses during sports activities, like heart rate variability and EEG patterns in athletes); (3) mental skills and psychological aspects in sports (stress management, anxiety reduction, and the mental state of athletes); (4) technological advancements in neurofeedback (the development of new EEG devices and their applications in sports); (5) ethical and methodological considerations in neurofeedback research (ethical implications, methodological challenges, and best practices in neurofeedback research); and (6) neurofeedback in specific sports (archery, golf, and shooting, examining how neurofeedback training can be tailored to the needs of athletes in these disciplines). Table 1 encapsulates the studies related to neurofeedback training and its application in sports performance, ranging from precision sports like archery and golf to considerations of psychological factors and technological advancements in neurofeedback application. Each entry aims to provide a succinct overview, synthesising the study’s objectives, methodology, key findings, and practical implications, offering a comprehensive view of the current state of NFT research within the sports science domain.

## 4. Neurofeedback and Target-Precision Sports

### 4.1. Theoretical Foundations

This section aims to clarify the theoretical underpinnings of neurofeedback’s application in precision sports, distinguishing it from earlier discussions by focusing on specific theories that explain neurofeedback’s effectiveness. It further elaborates on the contribution to understanding the role of neurofeedback in enhancing cognitive functions critical for target-precision sports, providing a deeper insight into the mechanisms behind neurofeedback’s impact on sports performance. The theories considered in neurofeedback and target-precision sports typically revolve around the concepts of brain plasticity, the psychophysiological model of biofeedback, and the attentional control theory. Brain plasticity underlies the idea that neurofeedback can lead to lasting changes in brain patterns associated with focus and calmness. The psychophysiological model emphasises how biofeedback helps athletes to become aware of and control physiological processes that influence performance. The attentional control theory relates to how athletes can improve their ability to maintain focus and avoid distractions during competition. These theories collectively explain how neurofeedback training might enhance the cognitive functions that are crucial for precision in sports.

### 4.2. Landers’s Early Studies

The theories outlined above paved the way for neuromodulation techniques to improve shooters’ specific abilities [56]. One of the first studies to use neurofeedback in archery was conducted by Landers in the early 1990s. The study aimed to determine whether EEG biofeedback training could improve sports performance by assessing aspects of concentration and confidence. The sample of 24 archers was randomly assigned to three treatment groups: a first group with correct feedback associated with increased low-frequency activity in the left hemisphere, a second group with incorrect feedback associated with increased low-frequency activity in the right hemisphere, and a third group with no feedback. The groups were pre-tested and post-tested on 27 firing trials. The study EEG data were collected from positions T3 (left hemisphere) and T4 (right hemisphere). The analyses showed significance only for sports performance, but not for concentration and confidence aspects. The group with correct feedback had significantly improved performance in comparison to the group with incorrect feedback, which instead showed a significant drop in shooting performance, while the control group showed no significant differences in performance before and after the test. This study provided the first concrete result on the use of EEG feedback as an effective means of providing performance improvements in elite archers [37]. Subsequently, Landers (1994) again highlighted the effects of learning with the electroencephalographic and electrocardiographic patterns of archers taking up the sport for the first time with the aim of verifying the cerebral hemispheric asymmetry and heart rate deceleration on task learning. At the end of 14 weeks, the archers had improved their performance by 62%, showing significant heart rate decelerations and EEG asymmetries between the best and worst shots at 12 Hz in the left hemisphere and 4 Hz in the right hemisphere [38].

### 4.3. Progress in Archery Research

Shooting sports are characterised by fine movements and a series of standardised, repeated actions that require a stable posture, along with adequate coordination and concentration and good psychological qualities [57]. An example is archery, where a clear mind, together with a high level of visual attention, allows the athlete to remain focused without external noise and internal mental distractions interfering with performance [40]. It has been seen that for professional archers, only 20% of their performance is determined by biomechanical factors, while the remaining 80% is solely attributed to psychological factors [58]. This is why the interest in studying these aspects has grown over time.

Kim conducted a study in 2014 in which he highlighted the brain activity of elite archers while comparing it with that of experts and novices. During the simulated archery activity, he identified the neural correlates of aiming performance by observing the differences according to the archers’ skill levels. The results showed that elite archers exhibited more activity in the supplementary motor area and cerebellum than experts and novices, and widespread but minimal activity in frontal areas involved in executive control, leading to the hypothesis that specialised neural processes may aid planning and motor control [44]. As abilities improve over time, certain processes mediate a reduction in electrical activity in executive control brain regions, causing a shift towards automated processing [43].

Recently, some authors investigated the differences in the neural and brain mechanisms of experienced and elite archers regarding motor behaviour, trying to identify a possible internal relationship between motor performance and brain activity. The authors collected and analysed the electroencephalography (EEG) results of 14 national and 14 provincial archers during aiming and resting phases. The results showed that elite archers had a stronger functional coupling in the beta1 and beta2 bands than experienced archers in the frontal and central regions, indicating a higher brain capacity for detailed movement control [50].

Previous research investigated hemispheric asymmetry and cardiac physiological response in the performance of elite archers to compare whether heart rate variations and hemispheric asymmetries would occur during aiming phases. The results showed that there was no deceleration of the heart rate during aiming and the alpha band was significantly greater in the left hemisphere than in the right. Furthermore, there were no significant EEG changes in the right hemisphere prior to firing and upon the release of the arrow [59]. Subsequently, similar studies were also conducted on experienced air-pistol shooters and found significantly higher alpha power at the left than at the right antero-temporal site after a comparison of shot quality [39].

### 4.4. Application of Neurofeedback in Shooting

Despite the years that have passed since Landers’s study on archers was published, little has yet been undertaken to clarify the role of neurofeedback in improving shooting performance skills. Chang and colleagues in 2020 conducted a review to understand the role of cortical activity in higher-order performance in precision sports tasks. Among the electroencephalographic components examined, only the sensorimotor rhythm showed a consistent and causal relationship with higher-order motor performance in precision shooting, whereas the results for left temporal alpha and frontal theta and alpha rhythms were not consistent [60].

A study prior to Chang’s theories indeed showed that higher sensorimotor rhythm power is associated with better performance in experienced shooters. The improved shooting performance of 24 shooters was associated with higher SMR power and lower alpha power at the Fz-T3 site before the start of a shooting action [46].

Recently, a similar investigation was conducted by Cheng and his collaborators involving two groups of shooters (upper group/experts and lower group/less experienced). The upper group showed increased accuracy and precision and reduced postural movements while aiming. EEG measurements revealed that the superior group showed a higher SMR rhythm before the shot compared to the inferior group, demonstrating reduced Fz-T3 coherence in shots taken in the centre of the target compared to missed shots. The researchers suggest that experienced shooters have a less strenuous pre-task attitude and less interference from non-essential cortical regions than less experienced shooters [42].

It stands to reason that shooters are required to focus on accuracy during competitions, and this pursuit of precision tends to be achieved by a peak sensory motor rhythm. For this reason, studies that have used neurofeedback in shooting sports have continued along this line. In 2012, Paul conducted a study on an experimental group of 28 university archers, who were asked to undergo an NFT protocol designed to amplify the SMR rhythm and inhibit theta rhythm for four weeks. After twelve training sessions, the archers in the experimental group were able to adjust their psychological state and EEG components during archery performance, demonstrating that an NFT protocol designed to reduce theta improves scoring regularity and accuracy [42].

Similar effects emerged in experienced air-pistol shooters through a bio/neurofeedback intervention with visual and auditory tasks. The authors concluded that a decrease in the theta wave and heart rate lead to improvements in attention and concentration. Furthermore, with an enhancement of the waves in the frontal regions and anterior cingulate gyrus, distracting moments can be reduced [47]. The practice of imagery, also known as visualisation, can also improve confidence, motivation, skill learning, and control in archery athletes [41]. Imagery flow can help shooting athletes set technical goals that are necessary for further growth. This technique is often used by the archer to familiarise themselves with the movements to be learned [61]. These states are associated with high theta activity in frontal areas and reduced alpha activity in frontal and central areas [62].

Salehi in 2019 found that both neurofeedback and motor imagery interventions were valid for training darts athletes. The study was conducted with 32 female dart shooters; the entire group was randomised into three intervention groups: (1) NFT intervention with a protocol designed to enhance theta and sensorimotor rhythms (SMR); (2) motor imagery, by means of internal (first-person) imagery, in which the participants had to imagine throwing darts; and (3) physical intervention, in which the participants physically practised throwing darts. The measurements of the three groups were recorded before and after the experimental intervention and compared to a control group. The findings showed that all three interventions significantly increased dart-throwing performance compared to the control group, demonstrating that, in addition to physical practice, both motor imagery and neurofeedback were found to be effective in improving performance in fine visualisation tasks [63].

Kavianipoor and colleagues, in 2023, sought to examine the implications of neurofeedback training on the executive control of attention and dart-throwing skills in female athletes with trait anxiety. The participant group consisted of twenty girls with an average age of 24.65 years, divided into two groups, one experimental neurofeedback group and one control group. All participants performed 14 training sessions. In the experimental neurofeedback group, training included SMR wave enhancement, theta wave presentation, and alpha wave enhancement, together with the dart-throwing exercise. In the control group, only the dart-throwing exercises were performed. After 48 h from the last training session, a post-test was conducted, which included a test of executive attention control networks (ANTs) and dart throwing. The results showed a significant difference in performance on both the executive control network and dart-throwing ability between the neurofeedback group and the control group. The results of the study confirmed the efficacy of neurofeedback training in improving the neural mechanisms of the executive control of attention and performance for throwing ability through enhanced attention processes [53].

Recently, a study examined the effect of theta wave power based on Frontal Midline Theta Power (FMT) training in elite biathletes [64]. The aim of the contribution was to determine whether neurofeedback training could increase FMT power and, consequently, shooting performance and attentional focus in biathletes. A sample of 28 biathletes were divided into two groups: a control group and an experimental group that received three hours of neurofeedback training. The authors observed that FMT increased during neurofeedback training compared to the initial value, verifying that neurofeedback was effective in temporarily increasing FMT, but no significant interactions were observed between the control and experimental groups in terms of shooting performance after training. The authors of the study suggested that training with neurofeedback temporarily increased FMT but did not lead to significant improvements in shooting performance. However, the results showed a small positive effect on attentional focus, and more experienced athletes showed that they were able to make the most of neurofeedback training to increase their FMT, but not their shooting performance [64]. A moment of distraction or a technical error can have a significant impact on the expected outcome, and mental preparation for managing anxiety in shooting sports requires impeccable concentration and consistency [59]. Mutang and colleagues recently conducted a study involving two archery athletes with the aim of testing the effectiveness of NFT in reducing anxiety symptoms and improving mental resilience in sport. The athletes in question underwent eight training sessions with neurofeedback to increase the sensorimotor rhythm (SMR) wave and inhibit high-frequency theta and beta waves in a specific brain area. Psychometric measures were used to assess anxiety, including the State-Trait Anxiety Inventory (STAI), the Sport Anxiety Scale-2 and the General Anxiety Disorder-7, along with measures of sports performance and mental resilience in sport. The authors found that both participants showed significant improvements in their mental resilience, including a greater sense of confidence, perseverance, and control, as well as that, thanks to this training, sport-related anxiety symptoms and general anxiety decreased significantly [54].

### 4.5. Enhancing Precision Sports Performance through Neurofeedback

In precision sports, maintaining an optimal psychological state during the pre-performance period is crucial to achieving peak performance. In precision sports such as putting and shooting in golf, motor programming is a fundamental psychological characteristic that organises and controls the many components involved in an action. Motor programming enables athletes to exercise appropriate motor control, such as force of movement, direction, and stability, to achieve superior performance [65]. Therefore, improving motor programming processes is essential to improve sport performance [66]. Previous electroencephalogram (EEG) studies have linked motor programming processes to the Mu sensorimotor rhythm (frequency of 8–13 Hz), particularly during motor preparation [67]. The literature suggests that increasing Mu power inhibits task-irrelevant motor programming processes, whereas decreasing Mu power facilitates task-relevant motor programming [68]. Studies have reported conflicting results regarding the relationship between Mu rhythm and visuomotor performance, possibly due to differences in skill levels and motor skill complexity studied. In golf, Mu rhythm was seen to reflect the allocation of cognitive resources to response motor programming during the observation and execution phases of direct actions during shooting [69]. The manipulation of Mu rhythm by means of electroencephalographic–neurofeedback (EEG-NFT) training offers the opportunity to explore its direct impact on performance. A single EEG-NFT session has been shown to alter EEG activity and improve performance in various tasks, including motor learning and sports performance [70].

A recent study conducted by Wang investigated the relationship between Mu rhythm and skilled performance in visuomotor tasks in golf, and how this relationship can be influenced by NFT. The study involved novice golfers who were divided into three groups: the decreased Mu rhythm group (DMG), the increased Mu rhythm group (IMG), and a dummy group (SG). The main finding indicated that the DMG experienced a significant reduction in Mu power after EEG-NFT, leading to better perceived control of their actions and better golf performance. This suggests a potential causal connection between Mu power and putting performance in golf, shedding light on the role of motor programming in the sport [71].

Golf, as is widely known, is a sport that requires a unique combination of physical skills, techniques, and mental qualities to excel, and neurofeedback can be a useful tool for golfers seeking to develop the mental and cognitive skills needed to perform better in their sport. However, the choice of neurofeedback training protocol, the location of electrodes, and the type of skills to be trained play an important role in influencing performance.

### 4.6. The Role of Motor Programming

Some studies have suggested that the enhancement of the sensorimotor rhythm (SMR) could indicate increased attention processing, leading to more effective putting in golf [45]. However, results are mixed, as some research has shown that neurofeedback with frontal alpha suppression in novice golfers did not lead to significant improvements compared to a control group [72]. Thus, further studies are needed to identify the most effective neurofeedback intervention to improve motor learning.

Recently, Afrash and colleagues, in 2023, sought to investigate the impact of NFT on motor learning in novice golfers [73]. The study involved 64 adult participants using three different NFT protocols: enhanced sensorimotor rhythm (SMR) in Cz, suppressed alpha waves in Fz, and suppressed Mu waves in Cz. The trial was divided into several phases, including a pre-test, an intervention (consisting of six sessions over two weeks), short-term maintenance (one day after the intervention), and long-term maintenance (two weeks after the intervention). During the intervention sessions, participants in the different groups received neurofeedback training based on one of the three protocols while also engaging in putting training. The results showed that there was no significant difference between the control and experimental groups during the acquisition phase. All groups showed a similar improvement in put accuracy. However, in the short-term retention phase, all three neurofeedback groups outperformed the control group. In the long-term retention phase, only the SMR and alpha groups showed better performance than the sham group, while the Mu group did not show significantly better performance. These results are in line with the existing literature and indicate the effectiveness of neurofeedback protocols based on SMR or alpha suppression in improving motor learning [49]. The authors argue that the positive effect of neurofeedback could be due to better regulation of the processes involved in motor programming and the creation of new brain connections through neuroplasticity, which improves memory, attention, and, consequently, motor learning. They also found that the brain waves recorded by experienced and novice individuals show significant differences, with smaller alpha waves and lower Mu waves in experts than in novices. These results suggest that neurofeedback can help modulate brain activities to improve motor performance [49].

### 4.7. Combined Training Approaches

Combining different training techniques can also achieve additive effects [74]. Neurofeedback and self-controlled practice could achieve combined positive effects on performance and motor learning in novice golfers. Pourbehbahani and colleagues, in 2023, examined the individual and combined effects of sensorimotor rhythm (SMR) neurofeedback and self-controlled practice on motor performance learning in novice golfers [75]. For this study, the authors involved 40 athletes, divided into four groups: neurofeedback/self-controlled practice, neurofeedback/controlled practice, sham practice/self-controlled practice, and sham practice/controlled practice. Self-controlled practice consisted of the participant being able to choose the colour of the ball (yellow, red, or blue); controlled practice required the participant to use a ball whose colour had been chosen by a member of the first group; sham practice characterised a sham group, whose participants were asked to watch a recorded video believing they could control the animation. Participants performed a series of putting trials on the green in four phases—pre-test, intervention, post-test, and follow-up—while SMR brain waves were recorded at a specific point (Cz). The results showed that although no positive interaction between the two practices was observed during the different phases of the experiment, both SMR neurofeedback and self-controlled practice independently improved putting performance (acquisition and post-test). However, only neurofeedback maintained its positive effects in the follow-up. Furthermore, neurofeedback was shown to increase the power of the SMR wave, regardless of the control type of practice. The same authors conclude that self-controlled practice, which allows participants to make choices even not strictly related to the putting task, improves motor learning. This effect could be attributed to an increase in motivation, the possibility to experiment with different strategies, and control over personal choices [75]. Giving trainees the opportunity to make decisions can improve learning and motivation, even when the choices are not strictly related to the training content [76].

### 4.8. Current Efficacies and Future Directions for Mobile EEG Technologies

In the sports literature, neurofeedback has been found to be beneficial in terms of motor learning, movement perception, learning speed, muscle strength, and fatigue [65]. But its effect on sports performance is still weak, as found in a meta-analysis evaluating the effect of NFT on sports performance and electroencephalography (EEG) power in athletes. Therefore, one should focus on developing new technologies and standardised neurofeedback protocols to ensure a more accurate assessment of the desired effects [9].

The development of new mobile EEG devices offers promising opportunities in the fields of exercise and sport [77,78]. Recently, the increasing use of consumer-level EEG (electroencephalography) devices has been verified over the last decade, and some authors have been able to verify that consumer EEG has proven to be a useful tool for neuroscientific research and that predictions provided by the latest studies predict massive use in the future as well [79]. Based on the study by Friehs et al. (2022), they point out that NFT, unlike brain stimulation, allows for more personalised and adaptive training, which is ideal for athletes who require in-depth monitoring of their cognitive functions [79,80].

There are many innovations in technology, and among the many devices concerning neurofeedback, the EEG-MUSE device is among the most innovative. This device is able to convert EEG signals into audio feedback to conduct neurofeedback sessions during sports training. In 2019, Raza and colleagues sought to further this knowledge by conducting a study involving eleven ten-pin bowlers, aged between 15 and 21, with the aim of determining whether the use of EEG-MUSE-based neurofeedback could improve performance and reduce anxiety in ten-pin bowlers [81].

They conducted a single 15 min neurofeedback training session in a quiet room as an intervention before the performance trials. Neurofeedback training (NFT) was provided using the MUSE EEG headset. Users received neurofeedback in the form of auditory signals such as wind and storm sounds. The intensity of the feedback increased with higher estimates of distraction and decreased with higher estimates of stability of attention. Before the start of the session, participants were able to choose a desired virtual environment in the app, such as rainforests or water waves, and then they could start the meditation session by pressing the start icon on the app. During the intervention, they were asked to remain as calm and relaxed as possible and to focus on their breathing. When the participant became distracted, they could hear the sound of the pre-selected environment changing, providing them with the feedback of self-regulation and concentration on breathing. In contrast, the control group received no intervention during the test period. The researchers could see from the results that those who received the neurofeedback intervention with MUSE scored slightly higher than the control group. But despite the trend, the data were not statistically significant. Moreover, the scores on the associated anxiety scale were also different between the two groups but not significant.

These findings are in line with the statements of Flanagan and Saikia (2023), who pointed out that consumer devices for EEG and fNIRS (near-infrared spectroscopy) are marketed for purposes such as relaxation, stress reduction, and attention enhancement, but are not intended for clinical or research use. Although some evidence suggests that neurofeedback may be useful in addressing disorders such as anxiety and attention disorders, the use of low-cost devices is still limited and further scientific evidence is needed [82].

In discussing the accessibility and application of neurofeedback for enhancing athletic performance, attention has been drawn to the emergence of consumer-grade, low-cost EEG (electroencephalography) devices. These devices have garnered interest for their potential to democratise the use of neurofeedback by making it more accessible financially. However, it is crucial to differentiate between the cost-effectiveness of these devices and the neurofeedback training process. While these devices offer a more affordable entry point into neurofeedback, the training protocols, expertise, and customization necessary for effective neurofeedback remain sophisticated and require professional oversight. The effectiveness of neurofeedback in sports performance enhancement hinges not just on the affordability of the hardware but on the quality of the training protocols and the skill of the practitioners. For this reason, we believe that continued investment in research is needed to implement new protocols that improve the training of athletes specialising in precision sports.

## 5. Technological Advancements for Neuromodulation and Biofeedback

### 5.1. The Significance of Neuromodulation in Enhancing Shooting Sports Performance

This review confirms the importance of induced neuromodulation in sports contexts, with a focus on target shooting sports. There is an increasing attention and care for performance details in shooting athletes. This is confirmed by the scientific community, which has long since consolidated the theoretical and practical bases of physical and mental aspects, thanks to training aimed at the growth of less-experienced athletes [83]. Studying in detail how an experienced athlete behaves from a mental point of view has allowed researchers to understand which direction to take in order to intervene in improving mental abilities [84]. Research on neuromodulation processes has found fertile ground on the improvement of these qualities and is identified as an effective support to be adopted in the athlete’s growth phases. The logic of this process is aimed at increasing performance, which is based on the identification of cortical activities and specific states or behavioural aspects deemed optimal.

It has been repeatedly demonstrated in the literature that neuromodulation training structured in micro-cycles of intervention of varying durations can induce momentary or permanent changes in the electroencephalographic tracing, resulting in improvements in intellectual, cognitive, emotional, and physical aspects, the dynamic combination of which plays a concrete role in the improvement of performance and the required skills [85,86,87].

### 5.2. From Landers’s Early Work to Contemporary Neuromodulation Strategies

Research began with these assumptions thirty years ago with the contribution of Landers who, not surprisingly, proposed an intervention with archers using neurofeedback/biofeedback aspects [37,38]. In recent years, progress has been made with findings that have seen a concrete and effective improvement in performance abilities, mostly treating physiological aspects [88]. For this reason, many studies have focused on physiological modulation, finding positive associations between physiological variables and the mental and performance aspects of shooters [89,90,91]. As far as neuromodulation is concerned, few studies have been presented and compared. In this regard, the results showed significant inconsistencies due to limitations regarding the sample, intervention methodology, and sport discipline [92]. For this reason, we believe that research must evolve by differentiating, with integrated protocols, both neurofeedback and biofeedback [93].

### 5.3. Synergistic Approaches: Combining Neurofeedback with Physiological Biofeedback

In exploring the synergistic effects of neurofeedback and biofeedback on athlete performance, it is critical to delineate the specific types of biofeedback employed alongside neurofeedback and their targeted physiological processes. Neurofeedback, focusing on the modulation of brain wave patterns through EEG signals, aims to enhance cognitive functions such as concentration and stress management. Concurrently, biofeedback encompasses a broader spectrum of physiological monitoring and training, including but not limited to heart rate variability (HRV) biofeedback for autonomic nervous system regulation, electromyography (EMG) biofeedback for muscle tension control, and galvanic skin response (GSR) biofeedback for emotional arousal and stress response management.

Integrating neurofeedback with HRV biofeedback can offer a holistic approach to optimising both mental and physiological states, fostering a psychophysiological coherence essential for peak performance. This combination is particularly advantageous in precision sports, where both cognitive focus and physiological calmness are paramount. Furthermore, EMG biofeedback can be strategically applied to sports such as archery and shooting, where muscle control directly influences accuracy and stability. By learning to manage muscle tension, athletes can achieve greater precision and steadiness. Additionally, incorporating GSR biofeedback can aid athletes in managing emotional arousal, ensuring that performance is not hindered by stress or anxiety.

This integrated biofeedback approach not only aims to enhance specific mental or physical aspects of athletic performance but also promotes a comprehensive improvement in the athlete’s overall ability to maintain optimal performance states under competitive pressure. By specifying and implementing distinct biofeedback modalities in conjunction with neurofeedback, our training protocols can be tailored more effectively to meet the unique needs of athletes in precision sports, thereby maximising their potential for high-level performance. An integrated approach would complete the intervention framework, shedding light on unresolved doubts and launching a procedure for new training. For this approach, research has found interesting insights into the technical act of shooting, showing more than satisfactory results [94,95,96].

### 5.4. Customising Neurofeedback for Enhanced Psychophysiological Consistency in Athletes

Based on the specific profiles of athletes, it has been suggested that those with a high capacity for self-regulation may benefit more from NFT, while athletes with lower sensitivity to biofeedback techniques may require complementary approaches [97]. Studies have shown that professional athletes exhibit different patterns of brain activity compared to beginners, indicating the potential relevance of individualised neurofeedback training for athletes [29]. Additionally, neurofeedback training properly adjusted to the athlete’s individual abilities has been found to impact psychophysiological consistency. Furthermore, the influence of noise or its absence on the performance of athletes and the success of NFT protocols has been highlighted, suggesting the need for further investigation in this area [30].

Moreover, it has been suggested that measures taken before and after stressors, based on the concept of autonomic response specificity, may be relevant in understanding athletes’ ability to self-regulate and their world ranking [98]. The athlete’s profile, which includes individual neurodynamic indicators, behavioural characteristics, and psychophysiological indicators such as EEG and heart rate variability, have been proposed as a valuable tool for understanding the functional state of athletes. Additionally, the use of biofeedback and neurofeedback with Olympic athletes has been discussed in the context of managing the stress response, emphasising the interconnectedness of the nervous, endocrine, and immune systems in the stress response [28].

Furthermore, the potential impact of neurofeedback training on athletes’ performance has been explored, with elite athletes serving as a model for understanding the effects of mastery, expertise, and skill execution [99]. Additionally, the results of a study on young football players suggest that correct feedback through neurofeedback can lead to improved performance, while incorrect feedback may reduce performance, highlighting the importance of precise feedback in neurofeedback training [100].

The literature suggests that individualised neurofeedback training may have a significant impact on athletes’ psychophysiological profiles and performance. The potential benefits of neurofeedback training for athletes with specific psychophysiological profiles, the influence of noise on performance, and the interconnectedness of various physiological systems in the stress response warrant further research to enhance our understanding of the application of neurofeedback in sports performance.

### 5.5. Exploring Neuromodulation’s Role in Athlete Recovery and Mental Well-Being

Future research should also investigate the impact of NFT on athletes’ recovery from injury and mental well-being, exploring how neuromodulation techniques can support resilience and psychological recovery. In exploring this potential use of neuromodulation, it is important to consider the current status of neuromodulatory therapies for consciousness disorders and the therapeutic application of neuromodulation in the human swallowing system [101]. These references provide insights into the potential applications of neuromodulation techniques in addressing neurological conditions, which could be relevant to understanding their potential impact on athletes’ psychological resilience and recovery.

In the context of neuromodulation, the promising role of neuromodulation in improving ischemic stroke recovery and the activity and neuromodulatory input contributing to the recovery of rhythmic output after decentralisation in a central pattern generator offer insights into the potential mechanisms through which neuromodulation may influence recovery and resilience in athletes [101,102].

### 5.6. The Expanding Horizons of BCI in Sports

Recent advancements in brain–computer interface (BCI) technology, particularly those utilising EEG signals, have significantly broadened the scope of neurofeedback applications beyond traditional mental and cognitive performance enhancement in sports. An exemplary demonstration of this technological evolution is highlighted by Pawuś and Paszkiel (2022). This study illustrates the sophisticated capabilities of EEG-based BCI systems, not only in interpreting cognitive states, but also in translating these states into actionable control commands for devices, such as wheelchairs, thus offering a new dimension to the practical applications of neurofeedback [103].

The integration of BCI technology in neurofeedback training (NFT) presents a unique opportunity to explore the multifaceted benefits of EEG beyond the realm of sports performance. By analysing EEG signals for power spectrum estimation and detecting specific patterns, such as nervous tics, BCI systems can provide real-time feedback and control mechanisms. This capability underscores the potential for EEG-based BCI to facilitate a deeper understanding of brain activity patterns associated with various cognitive states and motor intentions.

Incorporating BCI technology into the background of neurofeedback training emphasises the versatility of EEG as a tool for both performance enhancement and functional application. The success of BCI systems in accurately classifying EEG signals for practical purposes, such as wheelchair control, serves as a compelling example of how neurofeedback can extend beyond optimising mental skills in precision sports to include broader applications that enhance quality of life and functional independence.

## 6. Conclusions and Future Directions

The manuscript highlights the effectiveness of neurofeedback (NFT) in improving concentration, stress and anxiety management, and performance optimization in archery and precision sports athletes. The practical implication is that NFT can be used as a complementary tool in the training of these athletes to maximise their mental and cognitive abilities, positively affecting sports performance. This contribution reports studies that have applied neurofeedback protocols in precision disciplines such as archery and shooting. The results of the studies considered showed that neurofeedback can lead to faster reaction times, more sustained attention, and better emotion management, contributing significantly to athletes’ performance. Furthermore, it is emphasised that neurofeedback could be combined with other techniques such as motor imagination to maximise effectiveness in precision sports training. This review emphasises the importance of future studies that focus on the integration of neurofeedback with biofeedback in neuromodulation protocols. Current perspectives and limitations of research in this area are also indicated. Neuromodulation by means of neurofeedback represents a promising strategy to improve the overall mental and cognitive abilities of target shooting and archery athletes with an interesting potential for high-level performance. Future research should focus on integrated approaches and customised protocols to optimise the use of neurofeedback in a precision sports context. While our review provides valuable insights, it also highlights the need for more rigorous, controlled research in this area. Future studies, particularly randomised, controlled trials, are essential to establish more definitive conclusions about the effectiveness of neurofeedback in enhancing mental skills in precision sports. Future plans and observable trends within the field of NFT in precision sports incorporate the following points:

- Integration of NFT with Emerging Technologies

Future research should explore the integration of NFT with cutting-edge technologies such as virtual reality (VR) and augmented reality (AR). These technologies can simulate high-pressure competitive environments for athletes, allowing for the development of training protocols that not only enhance cognitive and mental skills but also prepare athletes for the psychological demands of competition.

- Personalisation and Machine Learning

An emerging trend is the personalisation of NFT protocols using machine learning algorithms. By analysing individual athletes’ EEG patterns and performance data, customised training programs can be developed that target specific areas for cognitive and mental improvement, potentially leading to more effective and efficient training outcomes.

- Wearable Neurofeedback Devices

The development of wearable and user-friendly neurofeedback devices will likely increase the accessibility of NFT for athletes. These devices could allow for more frequent and convenient training sessions, including the possibility of remote training, thereby expanding the use of NFT beyond the laboratory or clinic into daily training routines.

- Longitudinal Studies and Big Data

There is a need for long-term longitudinal studies that track the effects of NFT on athletes over multiple seasons or years. The use of big data analytics to process and analyse the vast amounts of data generated by these studies could uncover deeper insights into the long-term benefits of NFT and its impact on athletes’ careers.

- Interdisciplinary Research

Future plans should include fostering interdisciplinary research collaborations that bring together experts in neuroscience, sports psychology, cognitive science, and sports medicine. Such collaborations can lead to the development of holistic NFT protocols that not only improve specific mental skills but also consider the overall well-being and psychological health of athletes.

- Ethical and Regulatory Considerations

As NFT gains popularity in sports training, there will be a growing need to address ethical and regulatory issues, particularly concerning fairness, privacy, and the potential for misuse. Establishing clear guidelines and standards for the use of NFT in sports is crucial to ensure that it benefits athletes in a safe and ethical manner.

- Exploring New Sports and Disciplines

While much of the current research focuses on precision sports like archery and shooting, future studies should consider the application of NFT in a broader range of sports, including team sports and endurance sports, to investigate its potential benefits across different athletic disciplines.

In conclusion, the integration of neuromodulation and biofeedback into precision sports training represents a frontier of immense potential, offering targeted approaches to enhance athlete performance, mental resilience, and recovery processes. Through the meticulous application of neurofeedback and various biofeedback modalities, researchers and practitioners can tailor interventions that address the unique physiological and psychological needs of athletes. This personalised approach not only optimises performance in the short term but also contributes to the long-term development and well-being of athletes. Furthermore, the advent of brain–computer interface technology opens new avenues for applying these principles beyond traditional boundaries, promising innovative solutions for both athletic and therapeutic purposes. As we continue to explore the multifaceted benefits of neuromodulation and biofeedback, it is imperative to conduct further research to refine these techniques, validate their effectiveness, and fully realise their potential to transform the landscape of sports training and athlete care.

## Figures and Tables

**Table 1:** Relevant studies on neuromodulation, neurofeedback training, and precision sports.

Authors	Year	Topic	Precision Sports	Sample Description	NFT Protocol	Results Obtained
Landers, et al. [37]	1991	The influence of electrocortical biofeedback on performance in pre-elite archers.	Archery	Pre-elite archers	Electrocortical biofeedback	Enhanced performance and concentration
Landers, et al. [38]	1994	Effects of learning on EEG and ECG patterns in novice archers.	Archery	Novice archers	EEG and ECG biofeedback	Better learning outcomes and physiological control
Loze, et al. [39]	2001	Pre-shot EEG alpha-power reactivity during expert air-pistol shooting.	Shooting	Expert shooters	EEG alpha power	Higher accuracy in best vs. worst shots
Lee [40]	2009	Evaluation of attention and relaxation levels of archers using brain wave analysis.	Archery	Archers	Brain wave analysis	Improved focus and relaxation
Chang, et al. [41]	2011	Neural correlates of motor imagery for elite archers.	Archery	Elite archers	Motor imagery	Enhanced mental preparation and performance
Paul, et al. [42]	2012	Effect of SMR neurofeedback on psychophysiological measures and performance of archery players.	Archery	Archery players	SMR neurofeedback	Improved psychophysiological measures and performance
Callan & Naito [43]	2014	Neural processes distinguishing elite from expert and novice athletes.	Various	Athletes (various sports)	N/A	Differentiated neural processes by skill level
Kim, et al. [44]	2014	fMRI study of brain activity differences among elite, expert, and novice archers.	Archery	Archers (various skill levels)	N/A	Identified brain activity correlates of skill level
Cheng, et al. [45]	2015	Sensorimotor Rhythm Neurofeedback Enhances Golf Putting Performance	Golf Putting	Pre-elite and elite golfers	Sensorimotor Rhythm (SMR) Neurofeedback Training (NFT)	Improved putting performance and increased SMR activity
Cheng, et al. [46]	2017	Higher sensorimotor rhythm power associated with better performance in shooters.	Shooting	Skilled shooters	Sensorimotor rhythm neurofeedback	Improved shooting performance
Kakhaki & Taheri [47]	2017	Bio/neurofeedback training’s effect on performance, attention in elite shooters.	Shooting	Elite shooters	Bio/neurofeedback	Enhanced attention and performance
Mikicin, et al. [2]	2018	Effects of neurofeedback–EEG training on the level of attention and arousal control in sports shooters.	Shooting	Sports shooters	Neurofeedback–EEG training	Enhanced attention and arousal control
Christie, et al. [48]	2020	The effect of an integrated neurofeedback and biofeedback training intervention on ice hockey shooting performance.	Ice Hockey	Ice hockey players	Integrated neurofeedback and biofeedback	Improved ice hockey shooting performance
Gong, et al. [49]	2020	Efficacy, trainability, and neuroplasticity of SMR vs. alpha rhythm in enhancing shooting performance through neurofeedback training	Shooting	Students with basic pistol shooting skills	SMR (Sensorimotor Rhythm) vs. Alpha rhythm neurofeedback training	Improved shooting performance in the SMR group, decreased performance in the Alpha group
Gong, et al. [32]	2021	Review of neurofeedback training for sport performance from user experience perspective.	Various	Athletes	Various NFT protocols	Positive user experience and performance improvement suggestions
Chen, et al. [27]	2022	Effects of the function-specific instruction approach to neurofeedback training on frontal midline theta waves and golf putting performance.	Golf	Golfers	Function-specific instruction	Improved golf putting performance
Gu, et al. [50]	2022	Research on top archer’s EEG network topology from expert to elite.	Archery	Top archers	EEG network analysis	Insights into neural basis of elite performance
Cheng, et al. [51]	2023	QEEG markers of superior shooting performance in skilled marksmen.	Shooting	Skilled marksmen	QEEG analysis	Identified EEG markers linked to superior performance
Hatami, et al. [52]	2023	The effects of EEG-based neurofeedback training on learning air rifle shooting in novices.	Air Rifle Shooting	Novices in air rifle shooting	EEG-based neurofeedback	Enhanced learning of air rifle shooting
Kavianipoor, et al. [53]	2023	Neurofeedback training’s effect on attention and dart-throwing performance with anxiety.	Dart throwing	Individuals with anxiety	Neurofeedback	Improved executive control and performance
Mutang, et al. [54]	2023	SMR neurofeedback training on anxiety in archers.	Archery	Archers with anxiety	SMR Neurofeedback	Reduced anxiety and improved performance
Toolis, et al. [55]	2023	Neurofeedback training effects on shooting performance and attentional focus in biathletes.	Biathlon	Experienced biathletes	Neurofeedback	Enhanced focus and performance

“N/A” stands for “Not Available”.

## Data Availability

No new data were created or analyzed in this study. Data sharing is not applicable to this article.
